# DAILY SOCIAL RELATIONSHIP QUALITY, DAILY STRESS, AND SUBJECTIVE COGNITIVE DECLINE AMONG OLDER ADULTS

**DOI:** 10.1093/geroni/igad104.0364

**Published:** 2023-12-21

**Authors:** Heejung Jang, Jacqueline Mogle

**Affiliations:** Clemson University, Clemson, South Carolina, United States; Clemson University, Clemson, South Carolina, United States

## Abstract

Although prior research has shown that social relationships are strongly associated with cognitive function, few studies have explored the link between the quality of daily relationships and subjective cognitive decline (SCD). The present study explores whether the quality of daily relationships is associated with daily stress and how daily stress mediates the relationship between the quality of daily relationships and SCD among older adults. This study uses data from 254 older adults aged 70 or older who completed the Einstein Aging Study. Multilevel mediation analyses were conducted to account for daily measurements nested within individuals. The cognitive change index was used to measure SCD. We tested the indirect effect of daily relationship quality on SCD through daily stress levels. There was a significant positive association between ambivalent (β=.148, p<.001; β=.324, p<.001) and neutral (β=.095, p<.001; β=.297, p<.001) relationships and daily stress levels at both the within- and between-person levels. Between-person daily stress was, in turn, associated with greater SCD (β= .169, p<.05). In particular, there was a significant indirect path from ambivalent relationships to SCD through daily stress. This study contributes to a more detailed understanding of how relationship quality can impact cognition through increased exposure to daily stress. The quality of daily social relationships may be crucial to preserving older adults’ cognitive function.

